# Walking Speed: Japanese Data in Chronic Liver Diseases

**DOI:** 10.3390/jcm9010166

**Published:** 2020-01-07

**Authors:** Hiroki Nishikawa, Hirayuki Enomoto, Kazunori Yoh, Yoshinori Iwata, Yoshiyuki Sakai, Kyohei Kishino, Naoto Ikeda, Tomoyuki Takashima, Nobuhiro Aizawa, Ryo Takata, Kunihiro Hasegawa, Noriko Ishii, Yukihisa Yuri, Takashi Nishimura, Hiroko Iijima, Shuhei Nishiguchi

**Affiliations:** 1 Division of Hepatobiliary and Pancreatic Disease, Department of Internal Medicine, Hyogo College of Medicine, Nishinomiya, Hyogo 663-8501, Japan; enomoto@hyo-med.ac.jp (H.E.); mm2wintwin@ybb.ne.jp (K.Y.); yo-iwata@hyo-med.ac.jp (Y.I.); sakai429@hyo-med.ac.jp (Y.S.); hcm.kyohei@gmail.com (K.K.); nikeneko@hyo-med.ac.jp (N.I.); tomo0204@yahoo.co.jp (T.T.); nobu23hiro@yahoo.co.jp (N.A.); chano_chano_rt@yahoo.co.jp (R.T.); hiro.red1230@gmail.com (K.H.); ishinori1985@yahoo.co.jp (N.I.); gyma27ijo04td@gmail.com (Y.Y.); tk-nishimura@hyo-med.ac.jp (T.N.); hiroko-i@hyo-med.ac.jp (H.I.); nishiguc@hyo-med.ac.jp (S.N.); 2 Center for Clinical Research and Education, Hyogo College of Medicine, Nishinomiya, Hyogo 663-8501, Japan

**Keywords:** walking speed, handgrip strength, chronic liver diseases, ECW to TBW ratio, sarcopenia

## Abstract

We aim to clarify the impact of walking speed (WS) and analyze factors linked to WS decline in patients with chronic liver diseases (CLDs, 165 males and 191 females, 137 liver cirrhosis patients). The WS decline is defined as <0.8 m/second (m/s), referring to the guidelines. The median (range) WS was 1.3 m/s (0.2–2.02 m/s). There were 17 patients with WS < 0.8 m/s (4.8%). The WS value was significantly correlated with the handgrip strength value both in males (*r*^2^ = 0.252, *p* < 0.0001) and females (*r*^2^ = 0.256, *p* < 0.0001). In the multivariate analysis of factors associated with WS decline, only the extracellular water (ECW) to total body water (TBW) ratio using bioimpedance analysis was an independent predictor (*p* = 0.0398). Extracellular fluid excess was categorized as follows: normal condition (ECW to TBW ratio < 0.390), mild overhydrated condition (ECW to TBW ratio 0.390–0.399), and moderate to severe overhydrated condition (ECW to TBW ratio ≥ 0.400). The WS value was well stratified according to the ECW to TBW ratio (normal vs. mild, *p* = 0.0001; mild vs. moderate to severe, *p* < 0.0001; normal vs. moderate to severe, *p* < 0.0001; overall *p-*value < 0.0001). In conclusion, the ECW to TBW ratio can be closely linked to WS decline in CLD patients.

## 1. Introduction

Because sarcopenia, as defined by muscle mass decline and muscle strength decline in patients with chronic liver diseases (CLDs), can be associated with falls, decreased quality of life, or poor prognosis, it has gained much interest among clinicians these days [1,2,3,4,5,6,7]. Sarcopenia, especially, can occur frequently in patients with liver cirrhosis (LC), and it can be a risk factor for the development of hepatic encephalopathy in LC patients [8,9]. Japanese CLD patients are aging these days, and it is also an important public health problem as aging can be associated with sarcopenia [10,11]. How sarcopenia is associated with such adverse clinical outcomes needs to be looked at beyond the overt muscle mass loss or muscle strength decline and at this clinical entity as a systemic disease [12,13,14]. Muscle mass has been assessed by different techniques such as the bioimpedance analysis (BIA), dual-energy X-ray, computed tomography (CT) and magnetic resonance imaging, and the functional consequence of sarcopenia is estimable by handgrip strength (HGS) or other more composite tests such as the walking test [10,15,16,17]. Physical activity, nutrition, and control of underlying diseases may be required for the prevention of sarcopenia [5].

Walking speed (WS) can reflect skeletal muscle function and is an easy and reproducible measure [18,19,20]. Currently, the European Working Group on Sarcopenia in Older People (EWGSOP) criteria and the Asian Working Group on Sarcopenia (AWGS) criteria define 0.8 m/s as the cutoff point for WS decline [15,16]. However, in a population-based, cross-sectional survey of Japanese adults aged 65 years or older who were living independently at home in the community, the proportion of adults presenting a WS of < 0.8 m/s was only 3.6% (174/4811) [21]. The WS, thus, may not be suitable for the screening tool of muscle strength decline in Japanese older adults. Moreover, since clear consensus has not been reached with regard to assessment methods for WS (e.g., 12-, 10-, 8-, 6- meter walking), the Japanese Society of Hepatology (JSH) adopts only the HGS as a marker of muscle strength, and WS is not included in the current JSH guidelines for sarcopenia in CLDs [10]. Based on these backgrounds, there have been few reports examining the impact of WS in Japanese CLD patients [18,19]. In addition, recent research reported that in adults aged ≥60 years living in the community, WS decline and the HGS decline are associated with cognitive decline but there may be different mechanisms between brain and physical functions [20]. There is, thus, an urgent need for clarifying the impact of WS in CLDs, and in this study we seek to address these issues.

## 2. Patients and Methods

### 2.1. Patients

A total of 356 CLD individuals with data for WS were admitted to our hospital between July 2015 and January 2019, and they were subjected to this analysis. HGS data were available in all analyzed subjects, which were tested referring to the Japanese guidelines [10]. Presence of LC was judged by pathological findings (F4 or not), radiological findings (deformity of the liver, or the presence of varices or splenomegaly), and/or laboratory data (lower platelet count or lower prothrombin time) [22,23,24,25]. Mac-2 binding protein glycosylation isomer (M2BPGi) as a liver fibrosis marker was tested, as reported elsewhere [26]. Skeletal muscle index (SMI, kg/m^2^) was tested using BIA, as reported elsewhere [27]. SMI decline was defined as <7.0 kg/m^2^ in males and < 5.7 kg/m^2^ in females, referring to the Japanese guidelines [10]. Two patients had missing data for BIA. Severe ascites patients were not subject to this analysis owing to the limitation of BIA testing. We examined the relationship between the baseline of the WS value and clinical variables in a retrospective manner. WS decline was defined as <0.8 m/s referring to the current EWGSOP or AWGS guidelines [15,16]. Factors related to the WS decline were also explored in the univariate and multivariate analyses.

The current research protocol was approved by the institutional review board (IRB) of the Hyogo College of Medicine Hospital, and the declaration of Helsinki was strictly followed to ensure the rights of the research subjects (IRB approval no. 2296). Personal information was protected in the data collection.

### 2.2. Measurement of Walking Speed

In this study, a 6-m walking test was performed in each analyzed subject. We performed 6-m walking tests twice in each patient and adopted the average value of them as the walking speed (meter/second (m/s)).

### 2.3. Statistical Considerations

The JMP 14 software (SAS Institute Inc., Cary, NC, USA) was in use to do statistical analysis.

For the continuous parameter analysis, Student’s *t*-test, the Mann-Whitney U-test or Pearson’s correlation coefficient *r*, analysis of variance or the Kruskal–Wallis test was in use to adequately evaluate group differences. For the categorical parameter analysis, Fisher’s exact test or Pearson χ^2^ test was in use to evaluate group differences. Baseline items with significant correlation with the WS decline in our univariate analysis were subject to multivariate logistic regression analysis to choose candidate items. Unless otherwise mentioned, data were shown as the median value (range). The statistical significance threshold was set at *p* < 0.05.

## 3. Results

### 3.1. Baseline Features

**Figure 1:**
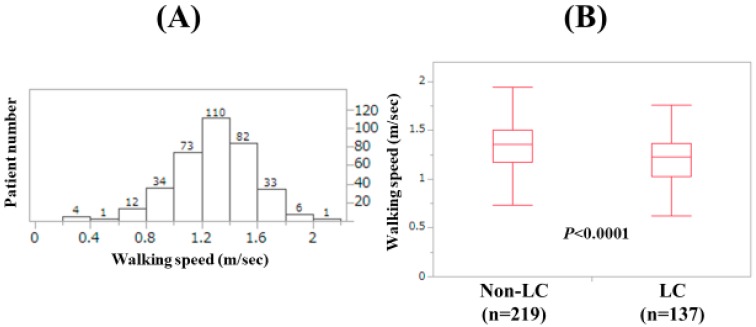
(**A**) Patient number distribution according to walking speed (WS). (**B**) The WS value according to the LC status.

**Figure 2:**
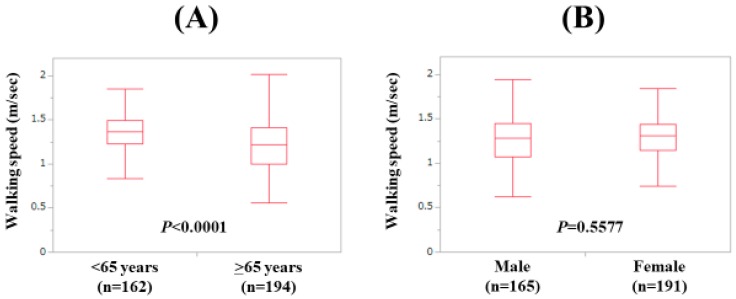
(**A**) The WS value according to age. (**B**) The WS value according to gender.

Baseline features of the analyzed subjects (n = 356) are presented in Table 1. The study cohort included 165 males and 191 females with the median age (range) of 66 (25–94) years. In terms of the disease etiology for CLD, hepatitis C virus was in the majority (181/356, 50.8%). The median (range) WS was 1.3 m/s (0.2–2.02 m/s). Number distribution for the WS value is demonstrated in Figure 1A. The number of patients with 1.2 m/s < WS < 1.4 m/s was the highest (110 cases, 30.9%). LC was identified at baseline in 137 cases (38.5%). There were 17 patients with WS < 0.8 m/s (4.8%) and 51 patients with WS < 1.0 m/s (14.3%). In males, the median (range) HGS was 33.6 kg (13.6–53.6 kg), while in females, the median (range) HGS was 20.5 kg (6.0–34.8 kg). Twenty-eight males (17.0%) and 57 females (29.8%) had the HGS decrease (HGS: <26 kg or <18 kg). In males, the median (range) SMI was 7.475 kg/m^2^ (5.21–11.01 kg/m^2^), while in females, the median (range) SMI was 5.875 kg/m^2^ (3.90–8.17 kg/m^2^). The median (range) WS in LC patients (1.23 m/s (0.33–1.76 m/s) was significantly slower than that in non-LC patients (1.36 m/s (0.2–2.02 m/s); *p* < 0.0001; Figure 1B). The difference of WS between patients aged <65 years (n = 162) and those aged ≥65 years (n = 194) also reached significance (*p* < 0.0001; median (range): 1.365 m/s (0.33–1.85 m/s) vs. 1.22 m/s (0.20–2.02 m/s); Figure 2A) The difference of WS between male patients and female patients did not reach significance (*p* = 0.5577; median (range): 1.28 m/s (0.2–2.02 m/s) vs. 1.31 m/s (0.33–1.84 m/s); Figure 2B).

### 3.2. Relationship between WS and HGS for All Cases, LC Cases and Non-LC Cases

**Figure 3:**
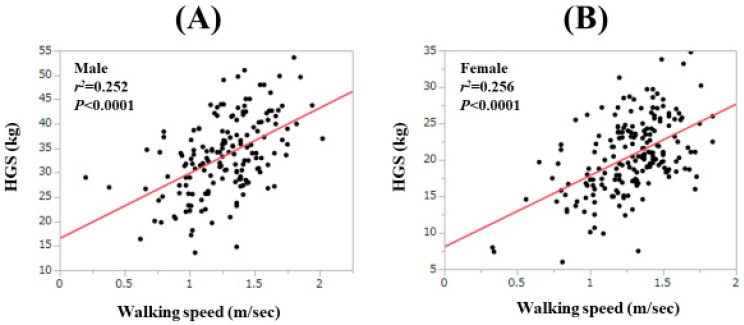
Correlation between the WS value and the handgrip strength value in males (**A**) and in females (**B**).

**Figure 4:**
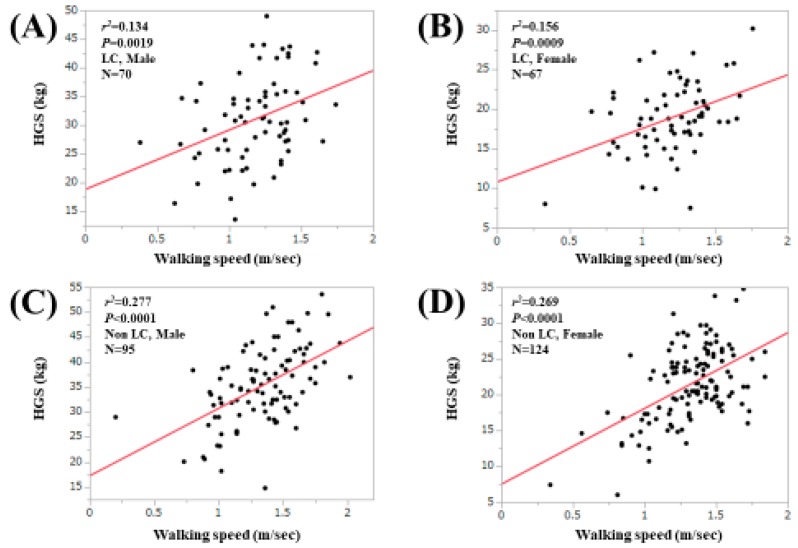
Correlation between the WS value and the handgrip strength value in male LC patients (**A**) and in female LC patients (**B**). Correlation between the WS value and the handgrip strength value in male non-LC patients (**C**) and in female non- LC patients (**D**).

For all cases, the WS value was significantly correlated with the HGS value both in males (*r*^2^ = 0.252, *p* < 0.0001) and females (*r*^2^ = 0.256, *p* < 0.0001; Figure 3A,B). For LC cases, the correlation between the WS value and the HGS value reached significance in both males (*r*^2^ = 0.134, *p* = 0.0019) and females (*r*^2^ = 0.156, *p* = 0.0009; Figure 4A,B). For non-LC cases, the correlation between the WS value and the HGS value reached significance in both males (*r*^2^ = 0.277, *p* < 0.0001) and females (*r*^2^ = 0.269, *p* = 0.0009; Figure 4C,D). In male patients, the proportion of patients with both HGS < 26 kg (cutoff value in the JSH guidelines) and WS < 0.8 m/s was 3.0% (5/165), while that with both HGS < 26 kg and WS < 1.0 m/s was 6.7% (11/165). In female patients, the proportion of patients with both HGS < 18 kg (cutoff value in the JSH guidelines) and WS < 0.8 m/s was 2.6% (5/191), while that with both HGS < 18 kg and WS < 1.0 m/s was 8.9% (17/191) [10].

### 3.3. Relationship between WS and SMI for All Cases, LC Cases and Non-LC Cases

**Figure 5:**
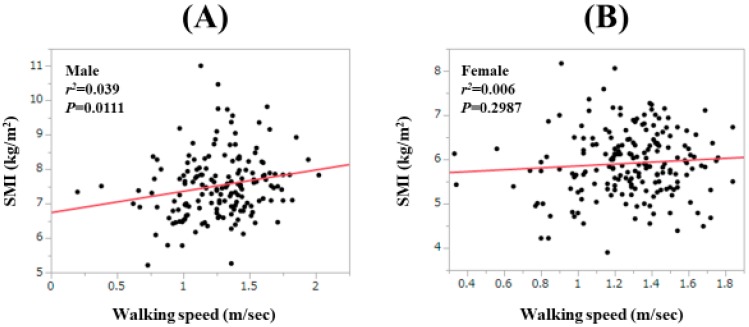
Correlation between the WS value and the SMI value in males (**A**) and in females (**B**).

**Figure 6:**
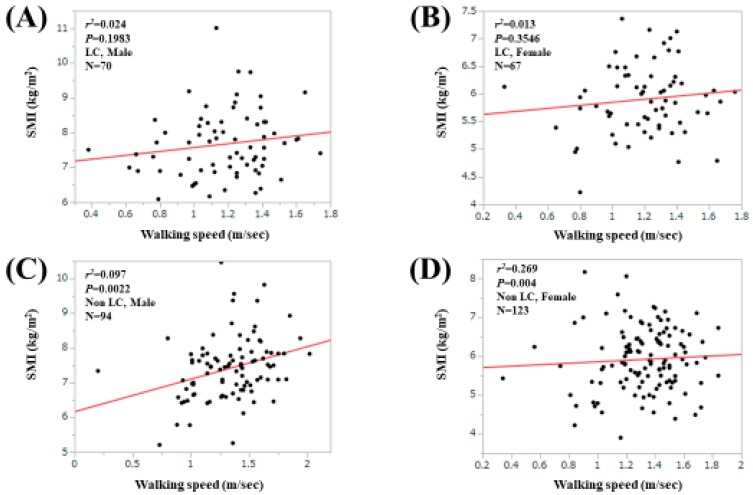
Correlation between the WS value and the SMI value in male LC patients (**A**) and in female LC patients (**B**). Correlation between the WS value and the SMI value in male non-LC patients (**C**) and in female non-LC patients (**D**).

For all cases, the WS value was significantly correlated with the SMI value only in males (*r*^2^ = 0.039, *p* = 0.0111; Figure 5A,B). For LC cases, the correlation between the WS value and the HGS value did not reach significance in both males (*r*^2^ = 0.024, *p* = 0.1983) and females (*r*^2^ = 0.013, *p* = 0.3546; Figure 6A,B). For non-LC cases, the correlation between the WS value and the HGS value reached significance only in males (*r*^2^ = 0.097, *p* = 0.0022; Figure 6C,D).

### 3.4. Correlation Coefficients between WS and Baseline Parameters Other than HGS

Correlation coefficients between WS and baseline parameters other than HGS are shown in Table 2. Among these, serum albumin involved the strongest positive correlation with WS (*r* = 0.309146, *p* < 0.0001), while the extracellular water (ECW) to total body water (TBW) ratio, as assessed by BIA, involved the strongest negative correlation with WS (*r* = −0.41169, *p* < 0.0001).

### 3.5. Uni- and Multivariate Analyses of Factors Related to the WS Decline (<0.8 m/s)

The univariate analysis found six variables to have significant association with WS decline (<0.8 m/s): age (*p* = 0.0278), presence of LC (*p* = 0.0088), serum albumin level (*p* = 0.0246), M2BPGi (*p* = 0.0141), branched-chain amino acid to tyrosine ratio (BTR; *p* = 0.0127) and ECW to TBW ratio (*p* < 0.0001; Table 3). Multivariate analysis for the six factors showed that only the ECW to TBW ratio (*p* = 0.0398) was a significant factor linked to WS decline (<0.8 m/s; Table 4). Hazard ratios (HRs) and 95% confidence intervals (CIs) are shown in Table 4.

### 3.6. Uni- and Multivariate Analyses of Factors Related to the WS Decline (<1.0 m/s)

The national center for geriatrics and gerontology in Japan (National Institute for Longevity Sciences-Longitudinal Study of Aging (NILS-LSA)) and the International Working Group on Sarcopenia (IWGS) define the optimal cutoff point of WS for muscle function decline as <1.0 m/s [17,28]. Thus, uni- and multivariate analyses of factors related to WS decline (<1.0 m/s) were also performed. The univariate analysis found seven factors to have significant association with WS decline (<1.0 m/s): age (*p* < 0.0001), presence of LC (*p* = 0.0124), serum albumin level (*p* = 0.0011), M2BPGi (*p* = 0.0016), BTR (*p* = 0.0289), ECW to TBW ratio (*p* < 0.0001), and presence of SMI decline (*p* = 0.0037; Table 5). In the multivariate analysis for the seven factors, no significant factor was found. Age (*P* = 0.0995) and ECW to TBW (*P* = 0.0556) tended to be significant. HRs and 95% CIs are shown in Table 6.

### 3.7. WS Stratified by ECW to TBW Ratio

**Figure 7:**
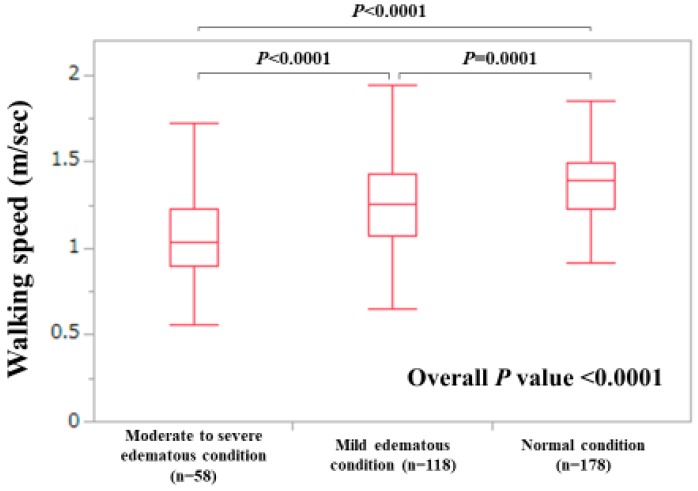
The WS value according to extracellular water (ECW) to total body water (TBW) as assessed by bioimpedance analysis. Normal condition indicates an ECW to TBW ratio < 0.390 (n = 178), mild overhydrated condition indicates an ECW to TBW ratio 0.390–0.399 (n = 118) and moderate to severe overhydrated condition indicates an ECW to TBW ratio ≥0.400 (n = 58). Missing data for the ECW to TBW ratio, n = 2.

Based on the results of multivariate analyses, we further examined the impact of the ECW to TBW ratio on WS decline. Extracellular fluid excess was categorized as the following three types: normal condition (ECW to TBW ratio < 0.390, n = 178), mild overhydrated condition (ECW to TBW ratio 0.390–0.399, n = 118) and moderate to severe overhydrated condition (ECW to TBW ratio ≥ 0.400, n = 58) [29]. As shown in Figure 7, the WS value was well stratified according to the ECW to TBW ratio (*p*-values: normal vs. mild, *p* = 0.0001; mild vs. moderate to severe, *p* < 0.0001; normal vs. moderate to severe, *p* < 0.0001; overall *p-*value < 0.0001).

## 4. Discussion

Physical activity has been considered a factor of interest in recent research. Previous data indicate that functional measures of muscle strength may have better predictability for outcomes than CT-based measures of skeletal muscle mass in LC patients [30]. As mentioned earlier, the current JSH guidelines for sarcopenia in CLDs adopt not WS but only HGS for the assessment of muscle strength decline and few research papers report data for WS in Japanese CLD patients [10,18,19]. Several reports have demonstrated that physical function precedes cognitive decline; however, the effects of HGS decline and WS decline on cognitive decline in adults may be different [20,31,32,33]. We, therefore, believe that to elucidate factors related to WS decline may be clinically meaningful.

In our data, the median (range) WS was 1.3 m/s (0.2–2.02 m/s) and there were only 17 patients with WS < 0.8 m/s (4.8%), which were in line with previous Japanese data [21]. In other words, as we expected, a small proportion of our study subjects had WS decline. In order to demonstrate the usefulness as a screening tool of WS for muscle strength decline, the cutoff value of WS < 1.0 m/s can be considered, as recommended by the NILS-LSA and IWGS, while the significant difference of the WS values between elderly and younger patients and that between LC and non-LC patients well reflected primary and secondary sarcopenia in CLDs [10].

The WS value was significantly correlated with the HGS value both in males and females for all cases, LC patients and non-LC patients, in this study. However, there are some cases where there was a gap between the WS value and the HGS value, as shown in Figure 3A,B. The improvement in WS requires quick movement, whereas the improvement in the HGS does not require it and this may account for our current results. Both maintained cognitive function and muscle strength may be necessary for the improvement of WS, while even if cognitive function is reduced, the HGS value will not decrease as long as muscle strength is maintained [20,34]. While the correlation coefficient between the WS value and SMI was not impressive compared with that between the WS value and the HGS value, muscle function and muscle mass may play different roles for the development of sarcopenia [19,30].

The ECW to TBW ratio is an independent factor linked to the WS decline (<0.8 m/s) and tends to be a significant factor linked to WS decline (<1.0 m/s) in our multivariate analysis. In addition, the WS value was well stratified according to the ECW to TBW ratio (normal condition, mild edematous condition, and moderate to severe edematous condition). The ECW to TBW ratio suggests extracellular fluid status (water homeostasis), including the whole body [29]. Excessive extracellular fluid may lead to the cognitive decline, and subsequent WS decline, which may be linked to our results [35,36].

In our previous investigation, we found that M2BPGi, a novel liver fibrosis marker, significantly correlated with the HGS value in both males (*r* = −0.4611, *p* < 0.0001) and in females (*r* = −0.33326, *p* < 0.0001) [37]. While in this study, the correlation coefficient between the WS value and M2BPGi was −0.2555 (*p* < 0.0001), which was slightly different from that between HGS and M2BPGi. In view of these results, the HGS and the WS may not be the same for the evaluation of muscle strength. However, future investigations will be necessary to confirm these results.

Frailty as well as sarcopenia has received increasing scientific attention as a potential explanation of the health diversity of elderly persons these days [38]. Frailty indicates a state in which physical activities have deteriorated due to age [39]. Decreased HGS, decreased WS, and decreased SMI can be associated with frailty [40]. In this study, the WS value was significantly correlated with the HGS value both in males and females and such tendencies were not observed in the correlation between WS and SMI. However, whether WS is more a sign of frailty or sarcopenia than HGS or SMI in CLD patients is unclear. Further research will be required.

Several limitations related to this study warrant mention. First, this was a single-center observational study with retrospective properties. Second, the study data was derived from population data on liver diseases in Japan, and additional studies on other races are needed to further validate and extend its application to other races. Third, the WS value can vary depending on various physical conditions. Fourth, large ascites patients who may be involved in WS decline were excluded because of the limitation of BIA, being the possibility of bias. Fifth, the effects of several diseases other than liver diseases that can affect WS (e.g., orthopedic diseases) were not fully examined. Finally, because of the cross-sectional nature of the data, the direction of association between baseline data and WS remains unknown. The results, therefore, must be interpreted with caution. Nevertheless, our study results indicate that the WS value in CLDs is well correlated with age or liver functional markers. Especially, an edematous state, as evaluated by BIA (i.e., the ECW to TBW ratio), was closely linked to WS decline. The HGS and WS should not be considered as the same assessment tool for muscle strength.

## Figures and Tables

**Table 1:** Baseline characteristics (n = 356).

Variables	Number or
	Median Value (Range)
Age (years)	66 (25, 94)
Gender, male/female	165/191
HCV/HBV/HCV and HBV/NBNC	181/61/9/105
Body mass index (kg/m^2^)	22.7 (14.6, 41.4)
Walking speed (m/s)	1.3 (0.2, 2.02)
Hand grip strength (kg, male)	33.6 (13.6, 53.6)
Hand grip strength (kg, female)	20.5 (6.0, 34.8)
Presence of LC, yes/no	137/219
Total bilirubin (mg/dL)	0.8 (0.2, 5.1)
Serum albumin (g/dL)	4.3 (2.0, 5.2)
Prothrombin time (%)	91.2 (11.9, 122.9)
Platelet count (×10^4^/mm^3^)	17.5 (1.4, 51.4)
FIB-4 index	2.16 (0.29, 30.98)
M2BPGi (cutoff index)	1.255 (0.21, 11.93)
SMI (kg/m^2^, male)	7.475 (5.21, 11.01)
SMI (kg/m^2^, female)	5.875 (3.90, 8.17)
ECW to TBW ratio	0.389 (0.367, 0.425)
AST (IU/L)	25 (10, 222)
ALT (IU/L)	19 (5, 232)
HbA1c (NGSP)	5.7 (3.7, 12.6)
eGFR (ml/min/1.73 m^2^)	81 (5, 173)
Serum zinc (μg/dL)	73.9 (22.0, 124.6)
BTR	5.65 (1.69, 13.82)
Serum ammonia (μg/dL)	38 (12, 206)

HCV: hepatitis C virus, HBV: hepatitis B virus, NBNC: non-B and non-C, HCC, LC: liver cirrhosis, M2BPGi: Mac-2 binding protein glycosylation isomer, SMI: skeletal muscle index, ECW: extracellular water, TBW: total body water, AST: aspartate aminotransferase, ALT: alanine aminotransferase, NGSP: National Glycohemoglobin Standardization Program, eGFR: estimated glomerular filtration rate, BTR: branched-chain amino acid to tyrosine ratio.

**Table 2:** Correlation between walking speed and baseline data other than handgrip strength.

	*r*	*p-*Value
Age	−0.3002	<0.0001
Body mass index	−0.02932	0.5825
Total bilirubin	−0.07285	0.1702
Serum albumin	0.309146	<0.0001
Prothrombin time	0.142872	0.0069
Platelet count	0.091789	0.0837
AST	−0.06229	0.2411
ALT	0.02578	0.6278
FIB-4 index	−0.17981	0.0007
HbA1c (NGSP)	−0.05046	0.3459
eGFR	0.090435	0.0884
SMI, male	0.189214	0.0149
SMI, female	0.091065	0.2127
ECW to TBW ratio	−0.41169	<0.0001
BTR	0.252886	<0.0001
Serum zinc	0.195912	0.0011
Serum ammonia	−0.16623	0.0039
M2BPGi	−0.2555	<0.0001

AST, aspartate aminotransferase; ALT, alanine aminotransferase; NGSP, National Glycohemoglobin Standardization Program; eGFR, estimated glomerular filtration rate; SMI, skeletal muscle index; ECW, extracellular water; TBW, total body water; BTR, branched-chain amino acid to tyrosine ratio; M2BPGi, Mac-2 binding protein glycosylation isomer.

**Table 3:** Univariate analyses of factors linked to walking speed (WS) decline (<0.8 m/s).

Variables	WS ≥ 0.8 m/s (n = 339)	WS < 0.8 m/s (n = 17)	*p-*Value
Age (years)	65 (29, 94)	71 (25, 83)	0.0278
Gender, male/female	155/184	10/7	0.3265
HBV/HCV/HBV and HCV/NBNC	60/170/9/100	1/11/0/5	0.4852
Body mass index (kg/m^2^)	22.7 (14.6, 41.4)	21.5 (17.7, 29.2)	0.3367
Presence of LC, yes/no	125/214	12/5	0.0088
Total bilirubin (mg/dL)	0.8 (0.2, 5.1)	0.9 (0.4, 3.2)	0.7231
Serum albumin (g/dL)	4.3 (2.0, 5.2)	4.0 (2.4, 4.6)	0.0246
Prothrombin time (%)	91.4 (11.9, 122.9)	84.7 (46.5, 103.9)	0.1597
Platelet count (×10^4^/mm^3^)	17.7 (1.4, 51.4)	16.0 (2.8, 32.2)	0.1401
AST	25 (12, 222)	27 (10, 191)	0.5570
ALT	19 (5, 206)	16 (5, 232)	0.2200
M2BPGi	1.24 (0.21, 11.93)	2.31 (1.08, 10.82)	0.0141
eGFR (ml/min/1.73m^2^)	81 (5, 173)	76 (7, 99)	0.0931
HbA1c (NGSP)	5.7 (3.7, 12.6)	5.7 (4.6, 7.2)	0.3573
Serum ammonia (μg/dL)	38 (12, 195)	48 (18, 206)	0.0534
BTR	5.71 (1.93, 13.82)	3.92 (1.69, 8.8)	0.0127
Serum zinc (μg/dL)	73.9 (22.0, 124.6)	68.4 (38.0, 94.4)	0.0764
ECW to TBW ratio	0.389 (0.367, 0.425)	0.398 (0.387, 0.421)	<0.0001
SMI decline, yes/no/unknown	114/223/2	7/10/0	0.6024

Continuous data are expressed as median (range). HCV, hepatitis C virus; HBV, hepatitis B virus; NBNC, non-B and non-C; LC, liver cirrhosis; AST, aspartate aminotransferase; ALT, alanine aminotransferase; M2BPGi, Mac-2 binding protein glycosylation isomer; eGFR, estimated glomerular filtration rate; NGSP, National Glycohemoglobin Standardization Program; BTR, branched-chain amino acid to tyrosine ratio; ECW, extracellular water; TBW, total body water; SMI, skeletal muscle index.

**Table 4:** Multivariate analyses of factors linked to the walking speed decline (<0.8 m/s).

	Multivariate Analysis		
	HR	95% CI	*p*-Value
Age ^#^	0.0267	9.251 × 10^−5^–7.700	0.1594
Presence of LC	0.779	0.109–5.589	0.8040
Serum albumin ^#^	15.152	0.0548–4184.100	0.3333
M2BPGi ^#^	0.764	0.0117–49.763	0.8998
BTR ^#^	8.007	0.0614–1044.325	0.3874
ECW to TBW ratio ^#^	0.00105	1.387 × 10^−6^–0.795	0.0398

^#^ When a continuous variable changes over the entire range. HR, hazard ratio; CI, confidence interval; LC, liver cirrhosis; M2BPGi, Mac-2 binding protein glycosylation isomer; BTR, branched-chain amino acid to tyrosine ratio; ECW, extracellular water; TBW, total body water.

**Table 5:** Univariate analyses of factors linked to walking speed (WS) decline (<1.0 m/s).

Variables	WS ≥ 1.0 m/s (n = 305)	WS < 1.0 m/s (n = 51)	*p-*Value
Age (years)	64 (29, 90)	73 (25, 94)	<0.0001
Gender, male/female	139/166	26/25	0.5446
HBV/HCV/HBV and HCV/NBNC	56/151/8/90	5/30/1/15	0.4421
Body mass index (kg/m^2^)	22.9 (14.8, 41.4)	22.4 (14.6, 30.6)	0.1246
Presence of LC, yes/no	109/196	28/23	0.0124
Total bilirubin (mg/dL)	0.8 (0.3, 5.1)	0.9 (0.2, 3.8)	0.9470
Serum albumin (g/dL)	4.3 (2.0, 5.1)	4.1 (2.4, 5.2)	0.0011
Prothrombin time (%)	91.4 (11.9, 122.9)	88.9 (33.4, 122.5)	0.1818
Platelet count (×10^4^/mm^3^)	17.9 (1.4, 51.4)	16.9 (2.8, 34.5)	0.1531
AST	25 (12, 222)	24 (10, 191)	0.3413
ALT	20 (6, 206)	17 (5, 232)	0.6215
M2BPGi	1.2 (0.21, 11.93)	2.18 (0.47, 10.82)	0.0016
eGFR (ml/min/1.73 m^2^)	81 (5, 173)	73 (7, 162)	0.0709
HbA1c (NGSP)	5.7 (3.7, 12.6)	5.7 (4.5, 9.8)	0.5730
Serum ammonia (μg/dL)	38 (12, 195)	40 (14, 206)	0.7299
BTR	5.715 (1.93, 13.82)	5.07 (1.69, 10.0)	0.0289
Serum zinc (μg/dL)	74.2 (22.0, 124.6)	69.5 (36.6, 118.2)	0.1474
ECW to TBW ratio	0.389 (0.367, 0.425)	0.398 (0.368, 0.421)	<0.0001
SMI decline, yes/no/unknown	94/209/2	27/24/0	0.0037

Continuous data are expressed as median (range). HCV, hepatitis C virus; HBV, hepatitis B virus; NBNC, non-B and non-C; LC, liver cirrhosis; AST, aspartate aminotransferase; ALT, alanine aminotransferase; M2BPGi, Mac-2 binding protein glycosylation isomer; eGFR, estimated glomerular filtration rate; NGSP, National Glycohemoglobin Standardization Program; BTR, branched-chain amino acid to tyrosine ratio; ECW, extracellular water; TBW, total body water; SMI, skeletal muscle index.

**Table 6:** Multivariate analyses of factors linked to walking speed decline (<1.0 m/s).

	Multivariate Analysis		
	HR	95% CI	*p*-Value
Age ^#^	0.0638	0.00205–1.989	0.0985
Presence of LC	0.973	0.320–2.957	0.9620
SMI decline	0.606	0.229–1.603	0.3132
Serum albumin ^#^	2.596	0.0989–6.817	0.5665
M2BPGi ^#^	0.887	0.0676–11.645	0.9275
BTR ^#^	1.121	0.0760–16.540	0.9337
ECW to TBW ratio ^#^	0.0284	0.000715–1.132	0.0556

^#^ When a continuous variable changes over the entire range. HR, hazard ratio; CI, confidence interval; LC, liver cirrhosis; SMI, skeletal muscle index; M2BPGi, Mac-2 binding protein glycosylation isomer; BTR, branched-chain amino acid to tyrosine ratio; ECW, extracellular water; TBW, total body water.

## Abbreviations

CLD	chronic liver disease
LC	liver cirrhosis
BIA	bioimpedance analysis
CT	computed tomography
HGS	hand grip strength
WS	walking speed
EWGSOP	European Working Group on Sarcopenia in Older People
AWGS	Asian Working Group on Sarcopenia
JSH	Japanese society of hepatology
M2BPGi	Mac-2 binding protein glycosylation isomer
SMI	skeletal muscle index
IRB	institutional review board
m/s	meter/second
ECW	extracellular water
TBW	total body water
BTR	branched-chain amino acid to tyrosine ratio
HR	hazard ratio
CI	confidence interval
NILS-LSA	National Institute for Longevity Sciences-Longitudinal Study of Aging
IWGS	International Working Group on Sarcopenia
